# Membrane Cascade Fractionation of Tomato Leaf Extracts—Towards Bio-Based Crop Protection

**DOI:** 10.3390/membranes13110855

**Published:** 2023-10-25

**Authors:** Emmanouil H. Papaioannou, Fabio Bazzarelli, Rosalinda Mazzei, Vasileios Giannakopoulos, Michael R. Roberts, Lidietta Giorno

**Affiliations:** 1 School of Engineering, Lancaster University, Lancaster LA1 4YW, UK; 2 National Research Council of Italy, Institute on Membrane Technology, CNR-ITM, Via P. Bucci, 87036 Rende, Italyl.giorno@itm.cnr.it (L.G.); 3 Lancaster Environment Centre, Lancaster University, Lancaster LA1 4YQ, UK

**Keywords:** biorefinery, membrane processes, tomato leaves, plant disease, bioactive compounds

## Abstract

Promising initial results from the use of membrane-fractionated extracts of tomato leaf as crop protection agents have recently been reported. This paper provides additional evidence from larger scale experiments that identify an efficient pipeline for the separation of tomato leaf extracts to generate a fraction with significant defence elicitor activity. A UF tubular membrane 150 kDa, with an internal diameter of 5 mm, proved appropriate for initial extract clarification, whereas afterwards a UF 10 kDa and three NF membranes (200–800 Da) in sequence were evaluated for the subsequent fractionation of this tomato extract. The compositions of sugars, proteins and total biophenols were changed in these fractions with respect to the initial extract. The initial extract ratio of sugars: proteins: biophenols was 1:0.047:0.052, whereas for the retentate of the 800 Da NF membrane, which has the higher crop protection activity, this ratio was 1:0.06:0.1. In this regard, it appears that the main crop protection effect in this fraction was due to the sugars isolated. It was found that with the appropriate membrane cascade selection (UF 150 kDa, UF 10 kDa and NF 800 Da) it was possible to produce (easily and without the need of additional chemicals) a fraction that has significant activity as an elicitor of disease resistance in tomato, whereas the remaining fractions could be used for other purposes in a biorefinery. This is very promising for the wider application of the proposed approach for the relatively easy formulation of bio-based aqueous streams with bio-pesticide activities.

## 1. Introduction

Agriculture contributes a substantial role to humanity, as it provides food, feedstocks and covers other human needs, such as clothing and construction components. Plants are the main convertors of the sun’s energy on earth, transforming and storing this energy chemically in plant biomass (~170 × 10^9^ tons/year), mainly in the form of carbohydrates (~75% *w*/*w*) [1]. When the same plants are cultivated intensively in large areas, then these are called crops and are of paramount importance for the economy and societies.

Tomato (*Lycopersicon esculentum*) is the second most cultivated vegetable crop, after potato [2,3,4]. Over the past decade, worldwide tomato production has been in the region of 180 million tons of fresh fruit per year [2,4] from ~5 million ha of harvested area. The tomato growing industry produces substantial amounts of waste streams from leaves and stems, as these are regularly removed from the vines to improve the fruit yield, as well as to reduce plant diseases [5,6,7,8]. In a study, the weight of branches and pruning leaves throughout the crop cultivation cycle amounted to ~0.16 kg per kg of tomatoes produced [6]. Based on this estimate, worldwide tomato production would generate ~28.8 million tons of this waste annually. This leaf material is currently used in anaerobic digestion systems or dumped in landfills [5,9]. However, anaerobic digestion downgrades valuable organic material and releases large quantities of CO_2_ into the atmosphere. Similar pictures emerge from other crop sectors growing crops in glasshouses. The agricultural sector has realised that other solutions are required to minimise the environmental impacts of these materials and create additional valuable products when feasible [10]. Tomato leaf waste streams are a rich source of phytochemicals, such as biophenols [1,8,11,12], solanesol [5], alkaloids [11,12], proteins [1,8] and carbohydrates [1], that could be better exploited and could have applications in many industrial fields [5,9,11,12]. Unfortunately, often the chemistry and biological activities of natural products derived from biomass varies according to crop cultivated [11], batch-to-batch variations, season and geographical location [13]. Such variations should be considered as a factor during bio-based product development.

Crop protection is considered crucial to the production of a safe and sufficient supply of agricultural commodities. Diseases can result in losses of up to 40% of the global crop yield every year [14,15,16]. Without effective crop protection, these losses could double [14]. To protect the crops, farmers use synthetic agents that inhibit or retard the growth of target microorganisms; however, the misuse of these products can result in the development of resistance and adverse environmental effects on other organisms and humans. In this regard, the use of natural products is gaining interest from farmers and governments, who are focusing on more sustainable production, reducing the risk of the development of pesticide resistance [16,17] and being more eco-friendly [18]. Tomato is prone to attack from more than 200 species of microorganisms [3]. *Pseudomonas syringae* is Gram-negative bacterium capable of infecting various crops [19] that has been extensively studied as a model pathogenic bacterium [20]. *P. syringae* infects plant tissues via wounds or natural openings in the leaf surface to colonise the intercellular space.

To cope with exposure to biotic and abiotic stresses, plants have evolved a variety of complex mechanisms of resistance. Many of these defence mechanisms are activated only when needed. Stress resistance responses are therefore regulated by diverse molecular monitoring systems that perceive internal and external environmental information, including specific stress-derived signals [21]. Many of these signals are molecules known as “elicitors”—compounds that are able to stimulate the plant’s own immune system. Plant defence elicitors are sub-divided into three main groups: pathogen-associated molecular patterns (PAMPs), herbivore-associated molecular patterns (HAMPs) and damage-associated molecular patterns (DAMPs) [22]. The use of these compounds for crop protection has been likened to the vaccination of plants [16].

There are many efforts to use extracts that act as elicitors from various natural resources, such as seaweed [23,24], extract of liquid vermicompost [25], tomato leaf [1], liquorice (*Glycyrrhiza glabra*) leaf extract [18] and other plant resources [3,17]. The elicitor activity in these cases has been attributed to proteins [23], polysaccharides [1,24], fragmented DNA [16], peptides [16], humic acid [25], biophenols [12], cell-wall fragments [16,26], etc. As plant-based extracts contain a variety of different compounds, they can synergistically simultaneously induce a number of plant defence mechanisms, thus being more effective than synthetic crop protection products [17,24]. Cell-wall fragments derived from pectins, such as oligogalacturonic acid (OGA) oligomers, have been demonstrated to induce defence-related changes [26]. Tomato leaves contain ~38 mg of pectin/g cell wall [27]. OGAs with a degree of polymerisation (DP) from 2 to 15 (molecular weight, MW, ~370–2658 Da) were tested in tomato plants by Simpson et al. [26], who found that those with DP 4–6 (MW ~722–1074 Da) were able to induce plant immunity. The isolation and purification of these OGAs is a quite tedious process that involves several stages and the use of chemicals and chromatographic purifications [26]. These quite complex purification procedures hinder the wider production of OGAs on an industrial scale, as they are quite expensive and lead to many side streams that have a negative impact on the environment.

Plant extracts are usually a mixture of compounds with different chemical characteristics and biological activities; thus, it is not always easy, necessary or economically viable to produce an extremely purified stream. In addition, some compounds in the extract may act synergistically to inhibit the growth of microorganism, something that is very important for avoiding the microorganism’s resistance. The vital role of membrane-based processes for producing streams in biorefineries enriched for specific compounds [1,28,29] and for recovering biologically active molecules from the agri-food sector has been extensively reviewed in the literature [10,30,31,32,33,34]. Membrane processes are widely acknowledged as unique in purifying and concentrating diverse solutions (such as juices, natural product extracts, etc.) and for separating valuable compounds from agri-food industry waste materials [10,33]. Nowadays, there is a growing attention on exploring and implementing environmentally friendly processes for recovering, purifying and concentrating bioactive compounds while keeping their activity intact. These processes aim to reduce the duration, the efforts and solvent consumption, while increasing the yield of bioactive compounds recovered. With this growing interest in natural compounds that possess biological activities, there has been a focus on their application in biophenol recovery from waste streams. Tight ultrafiltration (UF) and nanofiltration (NF) membranes have gained recognition for their ability to recover such biophenols [34]. In addition, membrane processes have been utilised for the refinement and/or concentration of oligosaccharide mixtures. For example, autohydrolysis liquors derived from discarded lemon peels were subjected to a dual-stage membrane filtration process involving diafiltration followed by concentration with the use of a regenerated cellulose membrane with a molecular weight cut-off (MWCO) of 1 kDa [35]. This process yielded a purified product containing roughly 98 wt% of oligosaccharides (DP 2–18) [35]. The separation efficacy of these membranes, as well as their productivity, hinges on several factors, including the membrane material, the MWCO and the operating conditions (such as pressure, temperature, feed flow rate, volume reduction factor, etc.) [34]. Membrane processes offer numerous advantages compared with traditional separation technologies, such as thermal and solvent-based processes. A key benefit is membrane processes ability to reduce separation costs, which can sometimes account for as much as 60% of the overall process expenses and comes from the phase transition included in other traditional processes, such as distillation and evaporation; the absence of this phase transition makes membrane processes less energy intensive [33]. The fundamental principle of membrane processes involves the use of a material barrier, typically made of polymers, ceramics or composites, to separate two different fluid streams containing the compounds to be separated. Membrane processes can achieve compound separation through various modes of operation, including pressure gradients, concentration gradients and externally applied fields [10]. Notably, these operations occur under relatively mild conditions and demand lower energy input compared with conventional methods while achieving the same level of separation or transformation. Membrane processes also offer scalability, automation, space efficiency and precise control in industrial settings [10]. A review of the literature showed that little attention has been paid to the fractioning of tomato leaf extracts by membrane processes. Only one previous study, [1], has investigated the suitability of integrated membrane processes for the production of bioactive fractions of tomato leaf extracts on a small laboratory scale (membrane area in the range from 12 to 42 cm^2^); this study indicated that the fraction containing components with molecular weights lower than 5 kDa was responsible for inducing plant defence against *P. syringae*. In the present work, the use of membranes was extended to a larger scale (membrane area of up to 690 cm^2^) and a larger gradient of pore size (from 150 kDa to 200 Da), further specifying the molecular size and nature of the components that act as “elicitors” in plant defence. These larger scale membrane processes allowed the study of higher volume reduction factors and have a better understanding of the biological activity of these fractions, something that is presented here for the first time.

## 2. Materials and Methods

### 2.1. Materials

All chemicals were purchased from Fisher (Fisher Scientific Ltd., Loughborough, UK) and were of analytical or HPLC grade, depending on the analysis or the process they were used for. The Folin–Ciocalteu phenol reagent, 2 M with respect to acid, was purchased from Sigma-Aldrich (Merck KGaA, Darmstadt, Germany).

Tomato plants were grown in a controlled environment growth room under fluorescent light (150 μmol∙m^−2^∙s^−1^ PAR, 12 h light, 12 h dark at 22 °C) at the Lancaster Environment Centre. The same plant growing conditions were used for the plants grown for the subsequent extraction, membrane fractionation and biological evaluation studies with membrane-derived fractions (Section 2.2.4). For the extraction, the plants were visually inspected; the defect free plants were cut and immediately homogenised with 1:2 *w*/*w* H_2_O:plant material with an ordinary blender, frozen at −80 °C, lyophilised and stored in sealed bags at room temperature in the dark until they were used to avoid any possible changes in composition. For the fractionation of biomolecules extracted from tomato leaves, commercial 30 cm long membrane modules were used sequentially from the higher to the smaller molecular weight cut offs (MWCOs). The technical details of the membranes are summarised in Table 1.

### 2.2. Methods

#### 2.2.1. Aqueous Extraction from the Tomato Leaves

**Figure 1:**
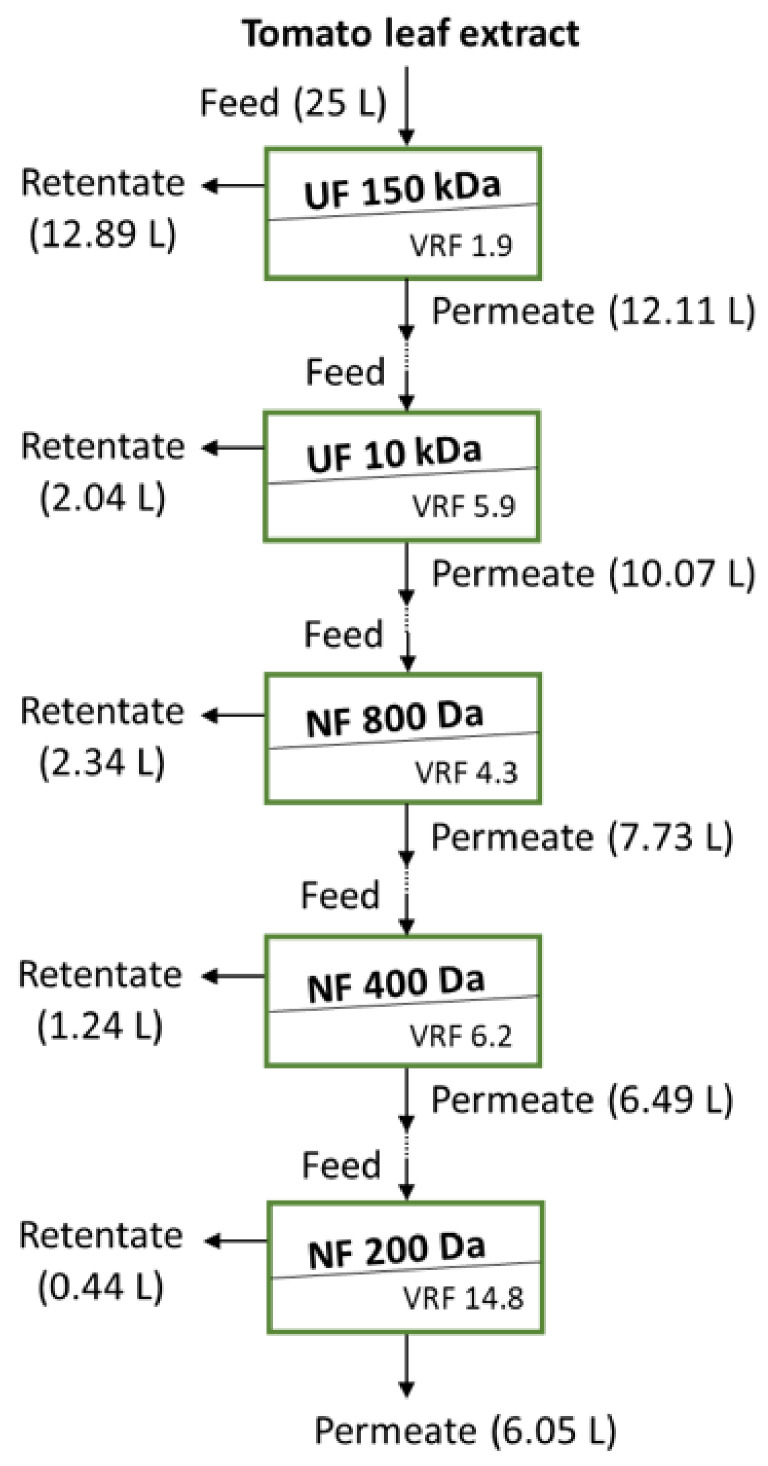
Schematic of the cascade membrane stages used to study the tomato leaf extract clarification and fractionation.

For the extraction of water-soluble active biomolecules, such as OGA, 1.25 kg of the tomato leaf powder was mixed with deionised water under the previously optimised conditions of a liquid-to-solid (L/S) ratio of 20 and a temperature 37 °C [1], whereas the pH in this larger scale extraction was not adjusted in order to avoid the production of an acidic stream. The initial pH of this stream was 6. The suspension was stirred at 300 rpm by an overhead stirrer (Pro40, SciQuip Ltd., Shropshire, UK) for 2 h, filtered through a 25 µm stainless-steel sieve (The Mesh company Ltd., Warrington, UK) and 0.2% *w*/*v* of potassium sorbate was added as a preservative. To avoid the influence of possible variation in the extract chemistry from batch to batch and prevent the extensive microbial growth for the evaluation of the UF membranes used for the initial clarification, 5 L was used in the feed vessel. The additional 20 L was kept in the fridge for a period of less than 3 days and added to this 5 L, thus giving a feed of 25 L (Figure 1) before use in the batch bleed concentration experiments with the most appropriate 150 kDa UF membrane. The clarified tomato leaf extract was used as feed for the UF 10 kDa (Figure 1).

#### 2.2.2. Membrane Process

The membrane processes used in this study are shown in Figure 1. The 150 kDa and 10 kDa UF membranes (Table 1) were used for the initial clarification (stage 1) and the removal of higher molecular weight compounds (MW) from the extract (stage 2), respectively, whereas the NF membranes (stages 3, 4 and 5) were used to fractionate the extract. As shown in Figure 1, the membrane processes were used in cascade, i.e., the permeate from each stage was used as feed for the subsequent stage; therefore, the permeate from the UF 150 kDa was fed to the UF 10 kDa, whose permeate was fed to the NF 800 Da, whose permeate was fed to the NF 400 Da, whose permeate was fed to the NF 200 Da. This evaluation of the various membranes helped to identify the main fractions with the desired biological activity and the initial operation conditions for the recovery of these compounds.

Each membrane module was placed in a stainless-steel holder and was tested under crossflow conditions. The membrane testing rig was composed of a jacketed thermostatic feed tank (30 L), a permeate tank and a progressive cavity pump (Seepex MD Pump Ltd., Somerset, UK). The pump flow rates were adjusted appropriately via a control panel. Temperature and pressure digital transducers were placed on-line after the membrane module, whereas a digital flowmeter was connected to the pump delivering site. The digital signals from these meters were continuously recorded during each experiment. The crossflow rates used in the UF stages were 84 and 87 L/h for the 150 and 10 kDa membranes, respectively (TMP: 1 bar), whereas the crossflow rate used in the NF stages was 87 L/h (TMP: 5 bar). The temperature during all experiments was maintained at 30 ± 2 °C. In case of the water membrane permeance measurements, the permeate flux was recorded continuously every 10 s for 20 min. During the treatment of the extract in batch concentration mode, the permeate flux was recorded continuously every 10 s until the desired volume reduction factor was reached (recycling the retentate stream to the feed tank and separately collecting the permeate).

Following the aqueous tomato leaf extract processing in each membrane filtration step, the fouled membranes were rinsed with deionised water at room temperature until the permeate was clear. Afterwards, the membrane was treated with NaOH (0.5% *w*/*v* aqueous solution) at 40 °C for 30 min, using TMPs of 1 and 5 bar for UF and NF membranes, respectively. Finally, the water permeance of membranes was measured at 30 ± 2 °C before and after the two cleaning steps to allow the calculation of the fouling index (FI) and cleaning efficiency (CE).

The FI was calculated using Equation (1).

(1) $$FI=\left(1-\frac{WP1}{WP0}\right)\times 100$$

where WP0 and WP1 represent the water permeance (L/h·m^2^·bar) of the membrane prior to and after the treatment of the tomato leaf extracts, respectively.

The CE was evaluated using Equation (2):

(2) $$CE=\left(\frac{WP2}{WP0}\right)\times 100$$

where WP_2_ is the water permeance of the membrane after NaOH cleaning.

The volume reduction factor (VRF) was calculated from Equation (3) and the batch concentration mode experiments

(3) $$VRF=\left(\frac{V_{f}}{V_{r}}\right)$$

where V_f_ and V_r_ are the feed and retentate volume, respectively.

The membrane % rejection (Rj) towards biomolecules in each processing stage was evaluated according to Equation (4):

(4) $$R_{j}=\left(1-\frac{C_{p,j}}{C_{f,j}}\right)\times 100$$

where C_p,j_ and C_f,j_ are the concentration of biomolecules in the permeate and feed stream of stage j, respectively.

An aliquot of 100 mL from the initial extract, the permeate, the retentate collected from each membrane processing step and the final permeate from the NF 200 Da were kept in the freezer until further analysis.

#### 2.2.3. Quantification of Biomolecules and Solids

Total solids (TS) and suspended solids (SS) dried at 103–105 °C were quantified using the 2540 B and 2540 D methods of the APHA (American Public Health Association), respectively, after the initial filtration through a 25 µm stainless-steel sieve. Briefly, for evaluation of the TS, a well-mixed sample was evaporated in a pre-weighed dish and dried to constant weight in an oven at 103–105 °C. The increase in weight over that of the empty dish represents the TS, according to Equation (5) below

(5) $$\mathrm{TS}\;\mathrm{mg}\;\mathrm{total}\;\mathrm{solids}/L=\frac{\left(A-B\right)\times 1000}{\mathrm{sample}\;\mathrm{volume}\;(\mathrm{mL})}$$

where: A = weight of dried residue + dish, mg, and B = weight of dish, mg.

For the SS, a well-mixed sample was filtered through a weighed glass-fibre filter (1.5 µm, Whatman, Cytiva, Marlborough, MA, USA) and the residue retained on the filter was dried to a constant weight at 103–105 °C. The increase in the weight of the filter represents the SS, according to Equation (6) below

(6) $$\mathrm{SS}\;\mathrm{mg}\;\mathrm{total}\;\mathrm{suspended}\;\mathrm{solids}/L=\frac{\left(A-B\right)\times 1000}{\mathrm{sample}\;\mathrm{volume}\;(\mathrm{mL})}$$

where: A = weight of dried residue + filter, mg, and B = weight of dried filter, mg.

The dissolved solids (DS) can be calculated from the difference between the TS and SS, according to Equation (7) below

DS mg total dissolved solids/L = TS − SS(7)

The total biophenol content (TBC) was quantified as gallic acid equivalent (GAE) at 750 nm by using the Folin–Ciocalteau assay [36] after modifying it slightly. In brief, 150 μL Na_2_CO_3_ was added into 1 mL of tomato leaf extract that had been diluted to be in the linear range of the calibration curve; 50 μL of Folin–Ciocalteau reagent was then added to this mixture. This mixture was left to react for 1 h at room temperature in the dark. The absorbance of the blue-coloured mixture was measured at 750 nm with a UV-Vis spectrophotometer (Evolution 220, Thermo Scientific, Waltham, MA, USA) versus the prepared blank (water containing the rest of the reagents). Nine gallic acid concentrations (3–30 mg/L) were used for the calibration. This method was employed to monitor variations in the concentrations of the total biophenols in each fraction rather than to determine the absolute concentrations of individual biophenols.

Proteins were quantified using the Bradford protein assay and bovine serum albumin (BSA) as a reference for the calibration (5–30 mg/L). The test was performed by mixing 1 mL of an appropriately diluted sample with 1 mL of Bradford reagent. The absorbance of the solution was measured at 595 nm after 30 min at ambient temperature.

An aliquot of 3 mL from each sample was stored at −20 °C until HPLC analysis; it was retrieved after defrosting it overnight in the fridge and filtered through a sterile 0.2 µm Corning PES membrane syringe filter. An amount of 0.1 mL of the filtered sample was diluted in a final volume of 1.4 mL with deionised water before HPLC analysis for sugars and for the molecular weight estimation. For biophenol analysis, 0.01 mL of the sample was diluted to 1.4 mL with water.

An Agilent 1260 LC system (Agilent Technologies, Santa Clara, CA, USA) with an Infinity II Quaternary gradient pump, vial sampler, multicolumn thermostat and refractive index (RI) detector was used for the analysis of sugars. The RI was operated at a stable temperature of 55 °C, and an analytical Hi-Plex H (300 × 7.7 mm, Agilent Technologies, Santa Clara, CA, USA) column was used. The different compounds were identified and quantified by comparing their retention times and responses with the standards (glucose, fructose and arabinose) in the range of 100–2000 mg/L. The mobile phase was 0.005 M H_2_SO_4_ in deionised water under a flow rate of 0.8 mL/min, and the column oven temperature was set at 60 °C.

For the molecular weight estimation, the same HPLC configuration was used with the SUPREMA (8 × 300 mm) column. The mobile phase was deionised water under a flow rate of 0.8 mL/min, whereas the column oven temperature was set at 40 °C in this instance.

For biophenol analysis, the HPLC system configuration was changed to include a DAD detector, whereas the RI detector was disconnected. A Poroshell 120 EC-C18 (3.0 × 150mm, 2.7 μm, Agilent Technologies, Santa Clara, CA, USA) column was used at 35 °C for biophenol analysis, using a slightly modified version of the method from Magiera and Zaręba [37], according to a binary gradient consisting of (A) 0.2% formic acid in water and (B) acetonitrile, as defined in Table 2. Spectral data from all peaks were accumulated in the range of 200–400 nm, and chromatograms were recorded at 280 and 325 nm. The different phenolic compounds were identified and quantified by comparing their chromatographic behaviour with the following external standards: at 280 nm: protocatechuic acid, p-hydroxybenzoic acid, rutin and salicylic acid; at 320 nm: chlorogenic acid, caffeic acid, ferulic acid, p-coumaric acid and sinapic acid in the range of 1–250 mg/L. Because the standards for all the compounds recorded in the chromatograms were not commercially available, they were determined in respect to the calibration of the two main groups of compounds at 280 and 320 nm, respectively.

#### 2.2.4. Biopesticide Activity of the Collected Fractions

Tomato plants were cultivated under the controlled environment conditions described in Section 2.1 for three weeks. After that period, an aqueous solution containing DS at a concentration of 1 mg/mL was sprayed onto the tomato leaves. This treatment was applied two days before the plants were infected with *P. syringae* to stimulate the plant’s immune response. The treated leaves were properly marked for subsequent infection. After the two days, the marked leaves were infected with *P. syringae* by immersing them for 20 s in a suspension containing 1 × 10^8^ CFU/mL prepared in a solution of 10 mM MgCl_2_, 0.2% *v*/*v* rifampicin and 0.05% *v*/*v* Silwet L-77. Following this infection, the plants were allowed to grow for an additional period of two days. Subsequently, the infected leaves were carefully removed from each plant, weighed and then homogenised in a mortar using a 1:10 *w*/*v* ratio with 10 mM MgCl_2_. From the supernatant of the homogenised leaf material, a 10 µL sample was taken and subjected to a ten-fold serial dilution. These diluted samples were then plated onto LB agar containing 0.2% *v*/*v* rifampicin. The plates were incubated for 30 h at 28 °C, and the resulting colonies were counted.

All experiments were performed in triplicate and expressed as the average value ± SD.

## 3. Results

### 3.1. Tomato Leaf Extract Analysis

The initial tomato leaf extract had average contents of dissolved solids (DS) and suspended solids (SS) of 14.5 ± 1.7 and 1.34 ± 0.15 g/L, respectively. The initial concentrations of biophenols and proteins in the tomato leaf extract were 580 ± 17 mg/L (TBC-GAE) and 518 ± 15.3 mg/L (Bradford assay), respectively. This protein concentration appears to be less than that predicted in the literature (~1250 mg/L [8], based on the protein concentration per gr of leaf), but can be explained by the fact that some proteins have remained in the solids fraction after the extraction. Thus, from the 14.5 g/L of DS, ~1.1 g/L corresponds to biophenols and proteins. The HPLC analysis shows that there were more than 40 phenolic compounds in the tomato extract (Figure S1); with a concentration of 423 ± 39 mg/L. These results are in good agreement with the TBC-GAE analysis. Because HPLC analysis quantification is based on well-known standards and their calibrations, it slightly underestimates the amount of total biophenols. The literature predicts a lower amount biophenols in tomato leaf extract (~300 mg/L [8], based on the biophenols concentration per gr of leaf). These differences in the contents of proteins and biophenols can be attributed to the different assay accuracies and/or tomato varieties and growing conditions; however, the values are of the same order of magnitude as those predicted in the literature. The concentration of sugars is 11.1 ± 0.7 g/L (based on the calibration for the main standards and the total area, Figure S2), thus the majority of the DS is made up of sugars. According to the literature [27], ~2 g/L of these sugars are considered to be of pectinid nature. GPC analysis shows at least 12 peaks (Figure S3). The highest MW detected was ~640 kDa and the lowest was ~400 Da. The higher response gave peaks with average MWs of 1.5, 2.3, 3.4, 4.7 and 15 kDa. These GPC results further support the initial membrane cascade selection. In the literature concerning tomato leaf proteins, molecular weights of 25, 37, 150, 175 and 250 kDa have been documented [17].

### 3.2. Fouling Index and Cleaning Efficiency in Cascade Membrane Processes

**Figure 2:**
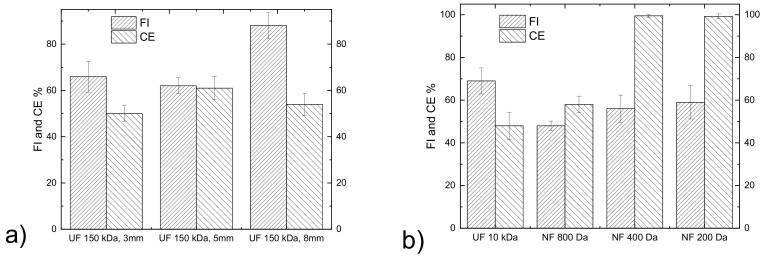
Fouling index and cleaning efficiency of membranes used in (**a**) the initial clarification of the tomato leaf extract and (**b**) for the fractionation of the clarified leaf extract.

Tomato leaf extract was clarified through UF 150 kDa membranes with inner diameters of 3, 5 and 8 mm. The same axial flowrate was used, and the axial velocities of these membranes were 27.5, 23.7 and 23.2 cm/s. As expected, SS were absent from the permeate for all three UF 150 kDa membranes tested. The FI and the CE of these membranes are presented in Figure 2a for the same VRF of ~2. The rejection and the recoveries of DS, proteins, biophenols and sugars were similar for these membranes, thus the 150 kDa UF membrane of 5 mm was selected for the clarification of the tomato leaf extract based on its slightly lower FI and higher CE. The clarified solution was sequentially treated, as explained in Figure 1, using a 10 kDa UF membrane and 800–200 Da NF membranes to create fractions enriched for compounds of different molecular weights. The aim of the first 10 kDa UF stage was mainly to remove the proteins and large MW polysaccharides, whereas the NF stages aimed to fractionate smaller MW polysaccharides and biophenols. The FI and the CE of the UF 10 kDa (VRF ~6) and NF 800 Da (VRF ~4) membranes are illustrated in Figure 2b. The FI of the 10 kDa membrane was higher than the NF 800 Da, likely due to the higher VRF (5.9 and 4.3, respectively). The CE for both the UF 10 kDa and 800 Da membranes was lower than the 400 (VRF ~6) and 200 Da (VRF ~15) NF membranes. This can be explained by the fact that the feed for the UF 10 kDa and 800 Da membranes had compounds with higher molecular weights that led to severe pore blockage. The smaller MWCO NF membranes have the same tendency to be fouled (Figure 2b) but their cleaning efficiency is close to 100%, which means that the fouling in this case is highly reversible and that the membrane performance can be easily restored by the gentle cleaning procedure that was applied.

### 3.3. Performance of Membrane Cascade and Characterisation of Tomato Leaf Fractions

**Figure 3:**
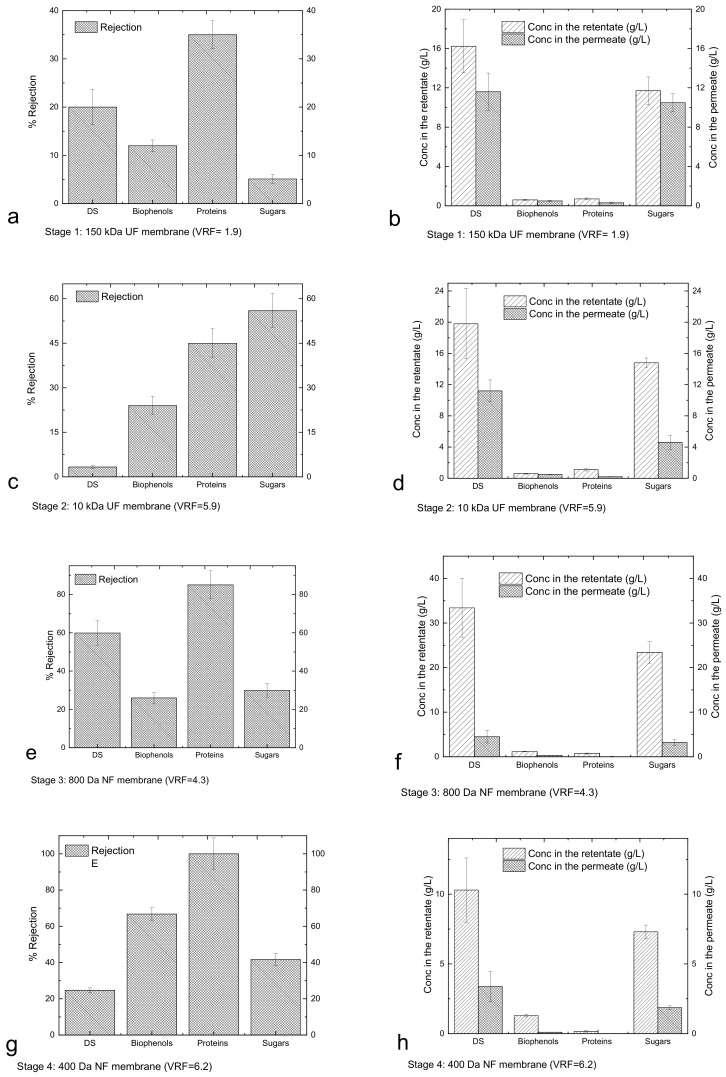

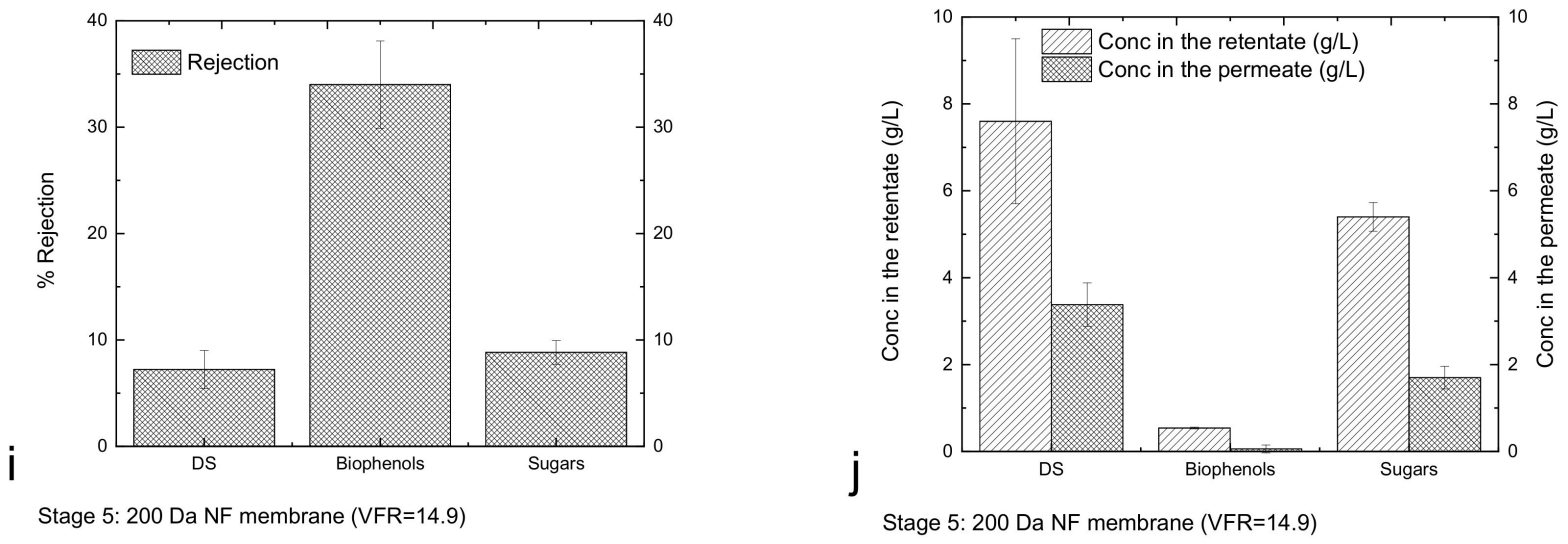
Rejection (Equation (4), (**a**,**c**,**e**,**g**,**i**)), and concentrations in the retentates/permeates (**b**,**d**,**f**,**h**,**j**) for each stage of the membrane cascade.

The performance of the membranes in each stage was evaluated in terms of the rejection coefficient for the four major categories of compounds of interest, whose concentrations in the retentates and permeates are reported in Figure 3.

Figure 3a shows that DS, proteins, biophenols and sugars passed through the UF 150 kDa membranes and were collected in the permeate. Since the membrane does not have selectivity for the mentioned components, their concentrations in the permeate are similar to those in the retentate (Figure 3b). The slightly higher concentration in the retentate reflects low rejection, most likely due to the concentration gradient at the membrane surface and the interaction of the components with the membrane leading to fouling. The use of the UF 10 kDa membrane (Figure 3c) led to a first fractionation of sugars, whereby fractions of carbohydrates with molecular weights higher and lower than 10 kDa were collected in the retentate and permeate, respectively (Figure 3d). Figure 3d also indicates that the DS are mainly constituted by sugars. Filtration through the NF 800 Da membrane (Figure 3e) promotes the significant fractionation of biophenols, proteins and sugars, resulting in a retentate enriched for components with molecular weights greater than 800 Da and a permeate containing components with molecular weights less than 800 Da (Figure 3f). The subsequent treatment through the NF 400 kDa membrane (Figure 3g) produces a retentate enriched for biophenols and proteins with molecular weights between 800 and 400 Da and sugars in the range of 800–400 Da and lower than 400 Da (Figure 3h). The NF 200 Da membrane (Figure 3i) produced a permeate enriched for sugars and biophenols smaller than 200 Da (Figure 3j). Cassano et al. [33] used a two-step nanofiltration process with spiral-wound membrane modules for the separation of biophenols from simple sugars (glucose, fructose and sucrose). The membranes used in that study were initially PES membranes with nominal MWCOs of 400 Da (1.8 m^2^) and then cross-linked aromatic polyamides with nominal MWCOs of 150–300 Da (2.6 m^2^). With this methodology, they were able to recover the most of biophenols with the first NF step, whereas the rejection of sugars was low. The second NF step that they used helped to recover the sugars and the remaining biophenols. In the recent study by Tonova et al. [29] evaluated the contribution of crossflow velocity and transmembrane pressure on the flux and rejection performance of a nanofiltration MWCO 500Da flat-sheet PES membrane (215 cm^2^) using Eurasian water milfoil hydrolysate. They found that they were able to obtain a glucose-rich permeate solution (54%) under an optimum TMP of 10 bar, whereas the retentate was enriched in other sugars and phenolics. The NF 200 Da membrane process used here had a hollow fibre configuration; it was made from modified PES and operated under 5 bar, so it was not possible to retain all the sugars that passed the NF 400 Da membrane. This can be explained by simple monosaccharide permeance. Therefore, the need for another material/configuration and/or appropriately tuning the operating conditions should be examined for recovering the remaining small sugars from the NF 400 or NF200 permeates. These small molecular weight sugars could have other applications in a biorefinery [38].

**Figure 4:**
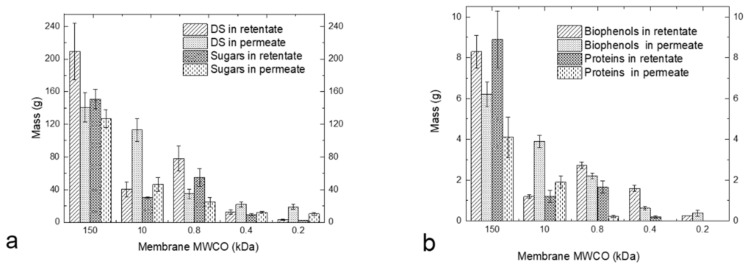
Mass (g) of DS and sugars (**a**) and mass of biophenols and proteins (**b**) in the retentates and permeates from different membranes.

Figure 4a shows the mass of DS and sugars in the retentates and permeates from different membranes, and Figure 4b shows the mass of biophenols and proteins in the same streams (it is worth noting that the high mass in the retentate of UF 150 kDa is due to the low VRF of this stage). The results confirm that sugars and proteins are mainly present in the fraction 10–0.8 kDa (retentate of 800 Da); biophenols with sizes between 400 and 800 Da are present in the retentate of 400 Da.

The proteins are retained from the larger MWCO membranes, leading to a permeate free of proteins after the 400 Da membrane. Cai et al. [31] previously used a similar methodology for fractionating the sugars, proteins and biophenols from *Hericium erinaceus*. They used a membrane cascade of MF 0.1 μm, UF PES 1000 Da and PA 300 Da under operating pressures of 4, 8 and 10 bar, respectively. With this cascade, they were able to alter the composition of the main components significantly, which is also the case in the present study. The membrane cascade described here is able to operate at lower pressures, e.g., 1 bar for the UF membranes and 5 bars for the NF membrane, which is advantageous from an energy perspective. In case of Cai et al. [31], some proteins passed the 1000 Da membrane and were present in the 300 Da retentate, whereas there is no evidence for this in the permeate in this process. In whey-derived peptide fractionation, Butylina et al. [39] were able to detect proteins (400 mg/L) in the permeate of the 1000 Da membrane, whereas in the present study the protein concentration in the NF 800 permeate was only 26.9 ± 6.8 mg/L.

### 3.4. Elicitor Activity of Tomato Leaf Fractions

For evaluating the biological elicitor activity of each fraction, initial experiments were conducted using a range of dilutions relative to DS concentrations (0.1–10 mg/mL) sprayed onto the tomato leaves. High DS concentrations were phytotoxic and caused visible “scorching” damage to leaves. Thus, the highest DS concentration that showed no visible toxicity (1 mg/mL) was used for the disease resistance assays. Herman et al. [18] mentioned that *Glycyrrhiza glabra* leaf extract at a concentration of 2% (*w*/*v*), which is equivalent to 20 mg/mL, resulted in *P. syringae* growth inhibition. Moo-Koh et al. [17] showed that the aqueous extract of *Croton chichenensis* roots at a concentration of 30 mg/mL resulted in inhibition of mycelial growth, sporulation and conidial germination against four fungi in vitro, whereas a concentration of 120 mg/mL was used in vivo in tomato plants in a greenhouse to mitigate the disease severity of the pathosystem *C. cassiicola*–*S. lycopersicum*.

Simpson et al. [26] used both commercial and purified trigalacturonic acid in the tomato plant bioassay at a concentration of 1 mg/mL. Both compounds had an elicitor-inducing effect at this concentration.

**Figure 5:**
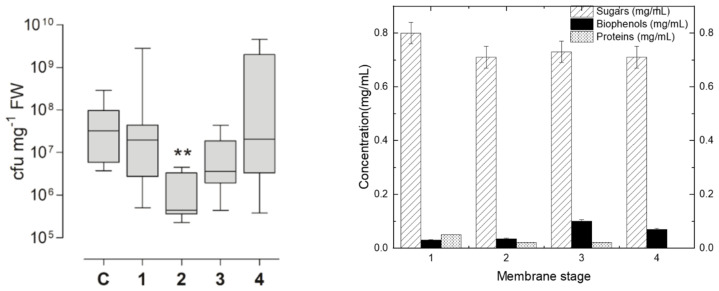
(**a**) Measurement of *Pseudomonas syringae* population growth (cfu: colony-forming unit) on tomato plant leaves after the “plant vaccination”, according to the protocol described in Section 2.2.4, with the use of the membrane produced fractions. Where C: control, 1: 10 kDa retentate, 2: 800 Da retentate, 3: 400 Da retentate and 4: 200 Da retentate. ** Denotes significantly different from control, *p* < 0.01 by Kruskall-Wallis test followed by Dunn’s multiple comparison post-hoc test. (**b**) The concentrations of sugars, biophenols, and proteins for each of the Figure 5a retentate fractions. These concentrations were calculated under the same final dilution of DS (1 mg/mL), based on each fraction’s initial concentration for each group of compounds.

The evaluation of the various fractions at a concentration of DS of 1 mg/mL for inhibiting the *Pseudomonas syringae* population growth on tomato plant leaves gave the results presented in Figure 5a. The biophenol, protein and sugar concentrations under this diluted 1 mg/mL final DS concentration are reported in Figure 5b, as they have been calculated in respect to their initial retentate concentration. The initial tomato leaf extract, the 150 kDa retentate and the 200 Da permeate gave similar results to the control. The retentate fraction obtained from the 800 Da membrane, which consisted of molecules with molecular weights lower than 10 kDa but higher than 800 Da, exhibited the highest inhibitory activity against the growth of *P. syringae* in tomato leaves (as shown in Figure 5a, labelled as number 2). This observed reduction in *P. syringae* growth (as depicted in Figure 5a, labelled as number 2) appears to be primarily attributed to the presence of sugars, rather than variations in the concentrations of other biomolecules, such as proteins and biophenols (as indicated in Figure 5b). Indeed, despite the higher concentration of biophenols in sample 3, there was no observed reduction in the growth of *P. syringae* (as shown in Figure 5a,b). Similarly, although sample 1 had a higher protein concentration, no decrease in *P. syringae* growth was observed (as depicted in Figure 5a,b). These findings highlight the suitability of fraction 2, the 800 Da retentate (composed mainly of molecules with molecular weights ranging between 10 kDa and 800 Da), for plant protection. As for the remaining fractions, they can be effectively directed toward other processes within a biorefinery.

## 4. Conclusions

The separation of bioactive compounds that can act as “elicitors” in plant defence was investigated through integrated membrane processes. Larger membrane areas were evaluated and a larger gradient of pore sizes (from 150 kDa to 200 Da) was used to identify the membrane fractions with the appropriate molecular size and the nature of the components that act as “elicitors” in plant defence. To achieve this, membrane rejection of the most important biomolecules (proteins, sugars and biophenols) was evaluated. In addition, growth inhibition studies using *Pseudomonas syringae* on tomato leaves were conducted using the biomolecules previously fractionated by the membrane processes.

The results of the membrane fractionation showed the levels of rejection of the sugars, which appear to be the main bioactive component in this case, for the 150 kDa, 10 kDa, 800 Da, 400 Da and 200 Da membranes were 5, 56, 30, 41 and 9%, respectively. The results showed that the retentate from the 800 Da membrane had higher biological activity against *P. syringae* infection due to the presence of sugars with the desired MW of oligosaccharides (in the range of ~722–1074), given the lower concentration in the other groups of compounds and given the inconsistencies of these compound concentrations with the observed biological activity results. Based on these results, the use of 150 kDa, 10 kDa and 800 Da membranes is sufficient to produce the targeted retentate of 800 Da, which has higher inhibition for *P. syringae* infection.

The utilisation of natural “pesticides” holds significant importance for crop protection. The methodology proposed herein enables the rapid and cost-effective production of formulations without the need for additional chemicals, thereby minimising adverse effects on the environment and human health.

Furthermore, the remaining fractions (e.g., the 10 kDa retentate and the 400 Da permeate) can be redirected to other added-value applications within a biorefinery, as they still contain substantial quantities of organic compounds. This approach aligns with the basic principles of the circular economy and provides additional revenues for otherwise waste material.

## Figures and Tables

**Table 1:** Technical details of the membranes employed for the tomato leaf extract fractionation.

Process	Material	Manufacturer	Configuration (Internal Diameter, mm)	Area (cm^2^)	MWCO (kDa)
UF	PES/PVP PVDF/PVP PVDF/PVP	Pentair	Hollow Fiber * (3), Tubular (5) Tubular (8)	430 180 170	150 150 150
	PES/SPES	Pentair	Hollow fibre (~0.8)	690	10
NF	Modified PES	NXFiltration	Hollow fibre (~0.7)	650	0.8
		NXFiltration	Hollow fibre (~0.7)	650	0.4
		NXFiltration	Hollow fibre (~0.7)	650	0.2

* The term hollow fibre, in general, refers to an internal tube diameter much lower than 1 mm. Here, the term “hollow Fiber” is used as commercial term, according to information provided from Pentair.

**Table 2:** Time scheduled gradient elution for the chromatographic analysis of the biophenols.

Time (min)	Mobile-Phase Composition		Flow Rate (mL/min)
	A (%)	B (%)	
0	98	2	0.4
3	87	13	0.8
6	87	13	0.8
9	85	15	0.8
11	80	20	0.8
13	55	45	0.8
16	25	75	0.5
19	80	20	0.5
21	98	2	0.4

## Data Availability

Data are contained within the article.

## Supplementary Materials

The following supporting information can be downloaded at: https://www.mdpi.com/article/10.3390/membranes13110855/s1, Figure S1: HPLC–DAD chromatogram of tomato leaf extract under the Table 2 gradient conditions, detection at 280 (A) and 325 (B) nm, Figure S2: Sugars chromatogram of tomato leaf extract (Refractive Index detector), Figure S3: GPC chromatogram of tomato leaf extract (Refractive Index detector).
