# Supplementary material for: Membrane Cascade Fractionation of Tomato Leaf Extracts—Towards Bio-Based Crop Protection
Source: Membranes (Basel). 2023 Oct 25;13(11):855. doi: 10.3390/membranes13110855 (PMC10673455; doi:10.3390/membranes13110855)
Supplement: Supplementary file 1 [file membranes-13-00855-s001.zip › membranes-2662744-supplementary.pdf]

Supplementary Materials:

Membrane cascade fractionation of tomato leaf extracts - towards bio-based crop protection

Emmanouil H. Papaioannou[1*], Fabio Bazzarelli[2], Rosalinda Mazzei[2*], Vasileios Giannakopoulos[3], Michael R. Roberts[3], Lidietta Giorno[2]

1 School of Engineering, Lancaster University, Lancaster, LA1 4YW, United Kingdom
2 National Research Council of Italy, Institute on Membrane Technology, CNR-ITM, via P. Bucci
3 Lancaster Environment Centre, Lancaster University, Lancaster, LA1 4YQ, United Kingdom
* Correspondence: e.papaioannou@lancaster.ac.uk (E.P.); r.mazzei@itm.cnr.it (R.M.)

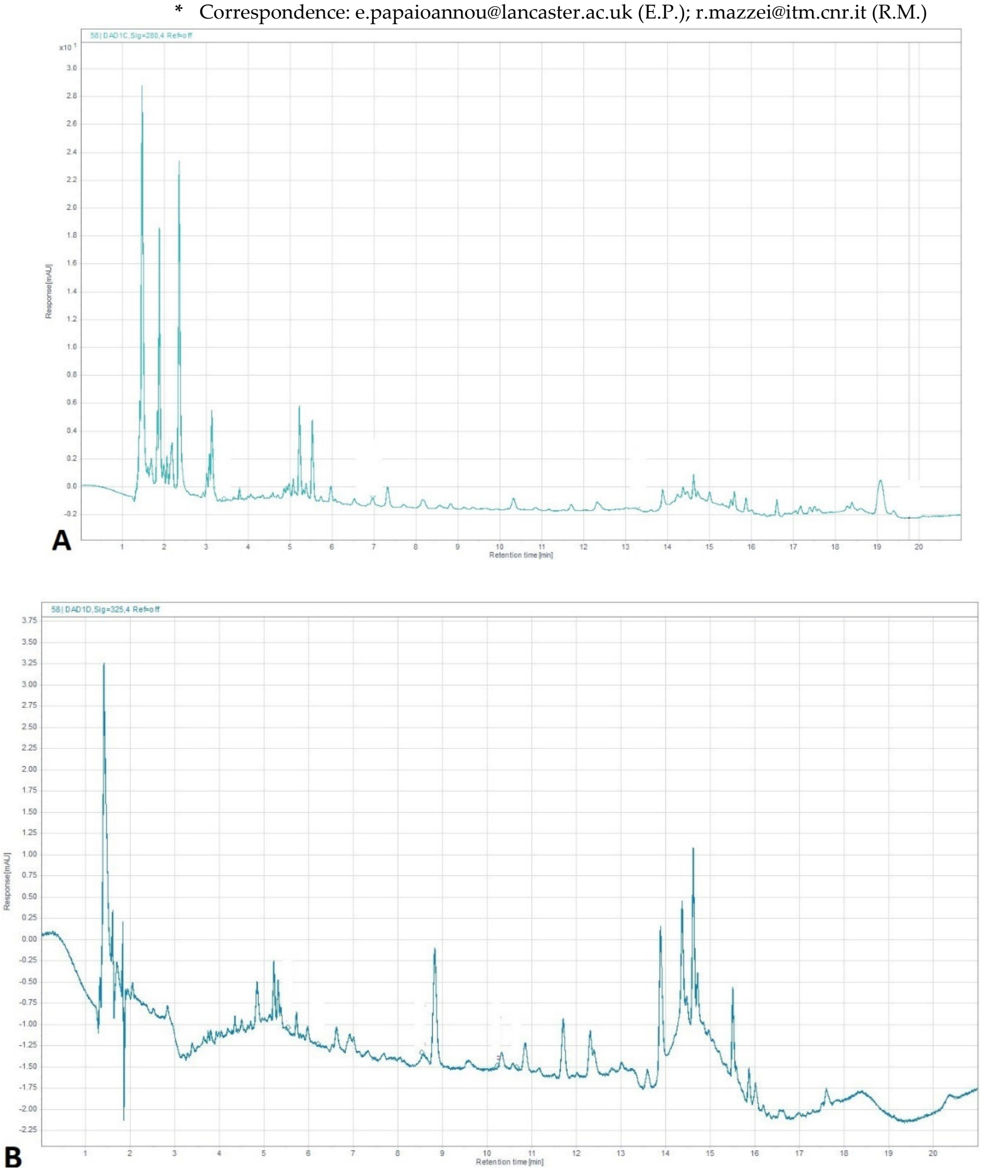

Figure S1 HPLC–DAD chromatogram of tomato leaf extract under the Table 2 gradient conditions, detection at 280 (A) and 325 (B) nm.

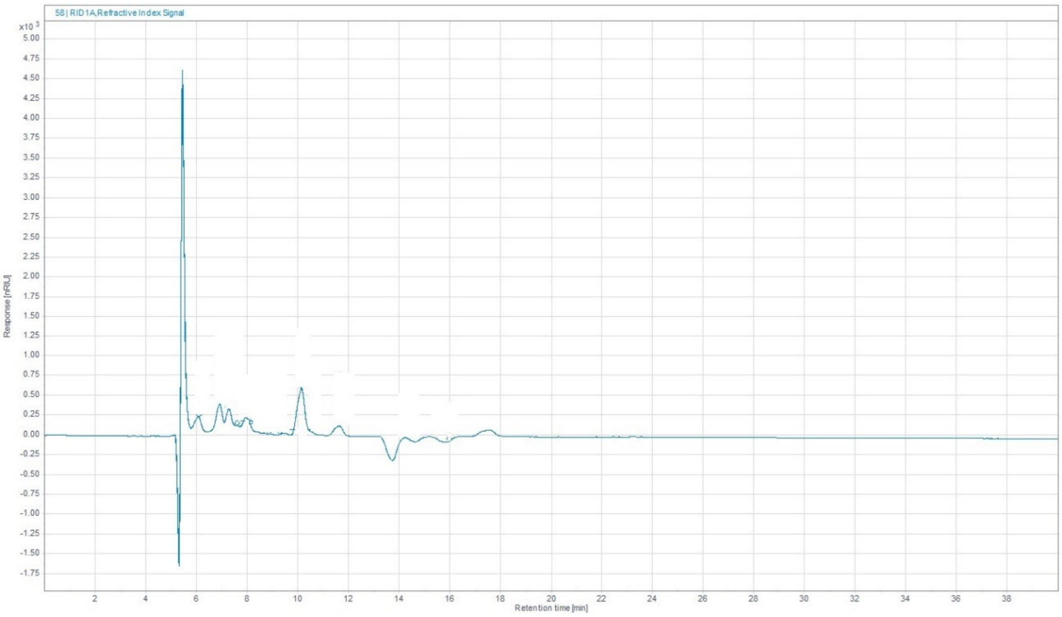

Figure S2 Sugars chromatogram of tomato leaf extract (Refractive Index detector).

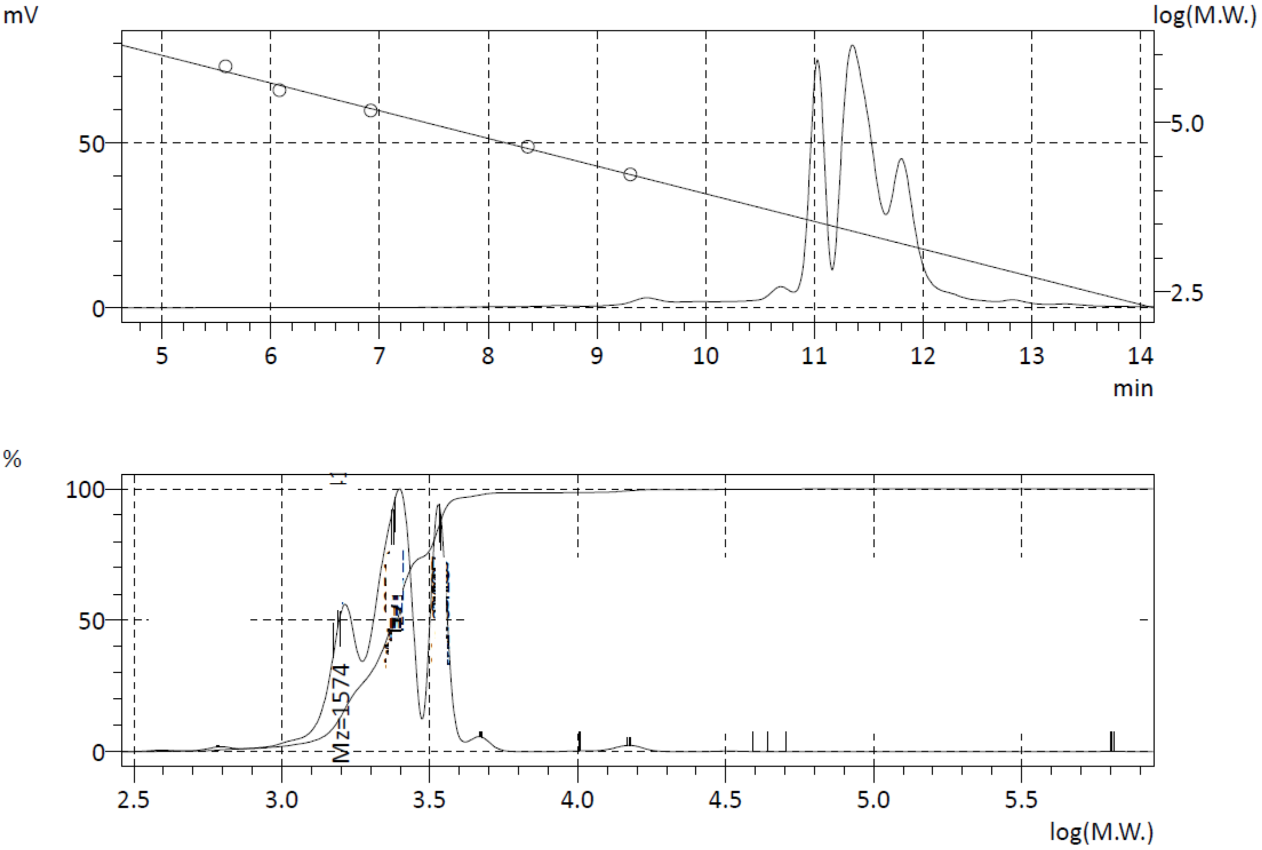

Figure S3 GPC chromatogram of tomato leaf extract (Refractive Index detector).
